# Effect of transport and rest stop duration on the welfare of conditioned cattle transported by road

**DOI:** 10.1371/journal.pone.0228492

**Published:** 2020-03-02

**Authors:** Daniela M. Meléndez, Sonia Marti, Derek B. Haley, Timothy D. Schwinghamer, Karen S. Schwartzkopf-Genswein

**Affiliations:** 1 Lethbridge Research and Development Centre, Agriculture and Agri-Food Canada, Lethbridge, Alberta, Canada; 2 Department of Ruminant Production, IRTA, Caldes de Montbui, Barcelona, Spain; 3 Department of Population Medicine, University of Guelph, Guelph, Ontario, Canada; Michigan State University, UNITED STATES

## Abstract

The effects of providing rest on physiological and behavioural indicators of welfare of cattle being transported by road has not been well studied in North America. New revisions to Canada’s *Health of Animals Regulations* Part XII: Transportation of Animals indicate un-weaned and weaned calves can be transported a maximum of 12 and 36 h, respectively, before an 8 h rest is required. Therefore, the aim of this study was to assess the effects of rest duration, after 12 and 36 h of transport, on physiological and behavioural indicators of welfare in 7–8 mo-old beef calves. Three hundred and twenty conditioned calves (258 ± 23.9 kg BW) were randomly assigned to a 2 × 4 factorial design where the main factors included transport duration: 12 h (**12**; *n* = 160) and 36 h (**36**; *n* = 160) and rest stop duration: 0 h (**R0**; *n* = 80), 4 h (**R4**; *n* = 80), 8 h (**R8**; *n* = 80) and 12 h (**R12**; *n* = 80). After the resting period, animals were transported for an additional 4 h. Blood and hair samples were taken from 12 animals per treatment prior to and after the first and the 4 h transport; and then 7 h, 2 d and 28 d after the 4 h transport. The concentrations of haptoglobin, creatine kinase, non-esterified fatty acids (NEFA), lactate, and serum and hair cortisol were determined. Standing and lying behaviour was assessed for 14 d after transport, while feeding behaviour of calves in one pen per treatment were assessed for 28 d after transportation using an electronic feed bunk monitoring system. Body weight (BW), average daily gain (ADG) and shrink (%) was assessed for all calves. The data was modeled using generalized linear mixed methods (SAS PROC GLIMMIX), where *transport* and *time* (nested in *rest*) were considered fixed effects and *animal* and *pen* were considered random effects. Statistically significant (*p* < 0.05) effects of transport were observed on BW and shrink, where 36 h-transported calves had lower (*p* < 0.01) BW and greater (*p* < 0.01) shrink than 12 h-transported calves. A transport × time (nested in rest) interaction (*p* < 0.01) was observed for lying percentage where, 36-R8 calves had greater (*p* < 0.01) lying percentage than 12-R8 calves on d 1 after transportation. The area under the curve (AUC) for NEFA was greater (*p* < 0.01) for 36-R0 calves than 12-R0, 36-R4, and 36-R8 calves, and greater (*p* < 0.01) in 36-R12 calves than 12-R12 calves. Haptoglobin AUC was greater (*p* = 0.05) in 36-R12 than 12-R12 calves. Overall, physiological indicators of reduced welfare were greater in calves transported for 36 than 12 h, while no clear differences were observed between rest stop groups with the exception of NEFA. Based on these results, conditioned calves benefit from shorter transport durations but there was no clear evidence that calves rested 4, 8, and 12 h following transportation experienced reduced transport related stress compared to those that were not rested (0h).

## Introduction

Cattle transportation is controversial from a welfare perspective, which has expedited the need to make changes to transport regulations. In North America, transportation of cattle is a vital part of the beef industry as ranches, feedlots and abattoirs are scattered across the country. Cattle can be transported 1 to 5 times in their lifetime and the ability to sell or buy cattle at lower or higher prices makes cattle transport across borders a common practice [1]. Handling, loading/unloading, comingling, trailer accelerations and vibrations, exposure to new environments, and extreme temperatures during transportation can cause stress [2,3]. Transportation and weaning stress have been reported to affect the immune response [4] increasing the susceptibility of calves arriving at the feedlot to diseases such as BRD [5,6]. Greater shrink during transportation has been reported in calves compared to market-weight cattle, suggesting that calves are more vulnerable to health problems, which can increase the incidence of morbidity and mortality for the first 30–60 d after their arrival at the feedlot, ultimately reducing calf welfare [7].

In the EU, weaned cattle can be transported for a maximum of 28 h when a 1 h rest period is provided after the initial 14 h of transport before a 24 h rest period is necessary [8], while in the USA, cattle are not allowed to be transported for more than 28 h prior to 5 h rest period [9]. The current Canadian transport regulations [10] state that ruminants can be transported for a maximum of 48 h before a 5 h feed, water and rest stop is required, unless the final destination can be reached within 52 h. Amendments to Canada’s *Health of Animals Regulation* slated to take effect February 20^th^ of 2020, state that un-weaned and weaned calves can be transported for a maximum of 12 and 36 h, respectively, before an 8-h rest period is necessary. Providing feed, water, and rest gives cattle an opportunity to recover from long-distance transportation, however, delaying the time to reach the final destination could increase stress due to the additional handling (loading/unloading), exposure to a new environment, and the risk of disease associated with exposure to pathogens [11].

Few studies have assessed the effects of transport and rest stop duration on indicators of cattle welfare and there is no research from which to provide science-based recommendations for the cattle industry or regulatory bodies. Therefore, the aim of this study was to assess the effects of rest stop duration after 12 and 36 h of transport, on physiological and behavioural indicators of welfare in 7-8-mo-old conditioned beef calves.

## Materials and methods

This protocol was approved by the Animal Care Committee of Lethbridge Research and Development Centre (ACC number 1816). Animals were cared for in accordance with the Canadian Council of Animal Care [12].

### Animal transport, housing and management

Three hundred and twenty newly weaned black Angus and black Simmental beef steers calves (mean ± standard deviation; 258 ± 23.9 kg of body weight (BW)) were transported on October 16^th^ 2018, for approximately 8 h from the ranch of origin to the Lethbridge Research and Development Centre. The day after arrival, calves received a 7-way bovine clostridial vaccine (Ultrabac/Somubac, Zoetis Canada Inc., Kirkland, Quebec, Canada); a 5-way bovine viral diarrhea, rhinotracheitis, parainfluenza and bovine respiratory syncytial virus vaccine (Pyramid FP 5 + Presponse SQ, Boehringer Ingelheim., Burlington, Ontario, Canada); an antibiotic (Draxxin, Zoetis Canada Inc., Kirkland, Canada); and an anti-parasitic agent (Ivomec Pour-on for Cattle, Boehringer Ingelheim, Burlington, Ontario, Canada). Ad libitum hay was provided the day of arrival to the feedlot, and a mix of 20% hay, 75% silage, and 5% minerals was provided on the second day. For the remainder of the experimental period calves received ad libitum feed consisting of 38.7% hay, 38.7% silage, 20% barley grain, and 2.5% supplement with vitamins and minerals to meet beef cattle nutrition requirements [13], as well as ad libitum water provided in a water trough located on the pen fence line.

Treatments consisted of a 2 × 4 factorial design where the main factors included transport duration: 12 h (**12**; *n* = 160) and 36 h (**36**; *n* = 160) and rest stop duration: 0 h (**R0**; *n* = 80), 4 h (**R4**; *n* = 80), 8 h (**R8**; *n* = 80) and 12 h (**R12**; *n* = 80). Calves were uniformly distributed by weight into 32 pens and calves were randomly assigned to pens. There were 40 calves in each treatment group, housed in 4 pens (10 animals/pen). Physiological and behavioural parameters were measured from 12 calves/treatment (3 calves/pen). The experimental calves (*n* = 320) calves were weighed and had their rectal temperature recorded. After the resting period, calves were transported, in the same trailers used for the initial transport, for an additional 4 h. A short transport duration was selected after the rest stop as we would expect cattle to be close to their final destination after a 36 h transport. Calves were divided into two groups of 160 calves, which were transported one week apart. The 36 h-transported and 12 h-transported calves in group 1 and group 2 were weaned, vaccinated, ear tagged, adapted to eat a grain diet from the feed bunk, and adapted to drink from the water trough in the feedlot pens (21 × 27 m) for 18, 19, 25, and 26 d prior to the start of the trial. Calves are “preconditioned” when weaning, castration, dehorning, branding, vaccination, ear tagging, and adaptation to a grain diet, the feed bunk and the water trough are completed 30 to 45 days before transportation [14,15]. Therefore, calves in the present study did not meet the criteria of preconditioning and they are referred to as conditioned calves.

Cattle were transported using model 379 Peterbilt trucks and 2018 Merritt feeder cattle tri-axle trailers bedded with wood shavings. Calves transported for 36 h left the Lethbridge Research and Development Centre at 1800 h on November 4^th^ 2018, and the 12 h group left at 1800 h on November 5^th^, and both trailers arrived at 0600 h on November 6^th^ (Tables 1 and 2). The following week, the second group of calves transported for 36 h left the Lethbridge Research and Development Centre at 1800 h on November 11^th^ 2018, and the 12 h group left at 1800 h on November 12^th^, and both trailers arrived at 0600 h on November 13^th^. During the 12 and 36 h transport, 80 calves were transported in the nose (*n* = 10), the deck (*n* = 30), the belly (*n* = 30) and the doghouse (*n* = 10) in each truck. During the additional 4 h transportation, calves from each rest group (*n* = 40) were transported in the nose (*n* = 10) and belly (*n =* 30). Calves were monitored by the truck drivers when they stopped for rest. If animals were lying down, the drivers got the calves to stand, to avoid injury. The same drivers transported the calves on two consecutive weeks, and the 36 and 12 h transports used the same route to ensure similar road conditions.

**Table 1:** Chronology of sampling for group 1 of conditioned black Angus and black Simmental calves transported for 12 or 36 h and rested for 0, 4, 8, or 12 h.

Samples	Group 1			
	0 h	4 h	8 h	12 h
LO1				
12 h	**Nov 4**^**th**^ 1537–1717			
36 h	**Nov 5**^**th**^ 1527–1701			
UN1	**Nov 6**^**th**^ 0546–0823			
LO2	**Nov 6**^**th**^ 0546–0627	**Nov 6**^**th**^ 1202–1236	**Nov 6**^**th**^ 1554–1628	**Nov 6**^**th**^ 2000–2040
UN2	**Nov 6**^**th**^ 1051–1131	**Nov 6**^**th**^ 1650–1727	**Nov 6**^**th**^ 2052–2129	**Nov 7**^**th**^ 0045–0132
7h	**Nov 6**^**th**^ 1755–1826	**Nov 6/7**^**th**^ 2351–0032	**Nov 7**^**th**^ 0352–0446	**Nov 7**^**th**^ 0755–0853
2 d	**Nov 8**^**th**^ 1052–1132	**Nov 8**^**th**^ 1655–1734	**Nov 8**^**th**^ 2058–2137	**Nov 9**^**th**^ 0059–0141
28 d	**Dec 4**^**th**^ 0807–1127			

Values indicate the date and time (24 h clock) sampling took place.

**Table 2:** Chronology of sampling for group 2 of conditioned black Angus and black Simmental calves transported for 12 and 36 h and rested for 0, 4, 8, or 12 h.

Samples	Group 2			
	0 h	4 h	8 h	12 h
LO1				
12 h	**Nov 11**^**th**^ 1522–1707			
36 h	**Nov 12**^**th**^ 1524–1705			
UN1	**Nov 13**^**th**^ 0530–0822			
LO2	**Nov 13**^**th**^ 0530–0611	**Nov 13**^**th**^ 1200–1234	**Nov 13**^**th**^ 1553–1631	**Nov 13**^**th**^ 1956–2026
UN2	**Nov 13**^**th**^ 1049–1126	**Nov 13**^**th**^ 1650–1729	**Nov 13**^**th**^ 2050–2124	**Nov 14**^**th**^ 0054–0124
7h	**Nov 13**^**th**^ 1755–1828	**Nov 13/14**^**th**^ 2354–0026	**Nov 14**^**th**^ 0354–0425	**Nov 14**^**th**^ 0755–0836
2 d	**Nov 15**^**th**^ 1057–1136	**Nov 15**^**th**^ 1653–1724	**Nov 15**^**th**^ 2053–2138	**Nov 16**^**th**^ 0100–0140
28 d	**Dec 11**^**th**^ 0811–1109			

Values indicate the date and time (24 h clock) sampling took place.

### Sample collection

Calves were sampled prior to loading (LO1) and after unloading (UN1) for the 12 and 36 h transports, and prior to loading (LO2) and after unloading (UN2) for the 4 h transport. In addition, animals were sampled 7 h and 2 d after UN2, and 14 d (weight and rectal temperature only) and 28 d after UN1. The R0 calves were unloaded and sampled (UN1) after 12 and 36 h transport, however these animals did not have a loading (LO2) sampling point prior to the additional 4 h transport because they were not rested. Prior to LO1 and LO2, animals were off feed and water for 2.5 h and 1 h, respectively.

#### Weight and rectal temperature

The experimental animals were weighed one-at-a-time while standing in a hydraulic squeeze chute (Cattlelac Cattle, Reg Cox Feedmixers Ltd, Lethbridge, AB, Canada) equipped with a weight scale and rectal temperature was collected using a digital thermometer at LO1, UN1, LO2, UN2 and 7 h, 2, 14, and 28 d after UN2. Average daily gain (ADG) was calculated by subtracting the final (d 28) and initial (LO1) BW and dividing it by the number of days on trial (36 h group: 31 d; 12 h group: 30 d). Shrink percentage was calculated for the 12 and 36 h transport (shrink 1) and the additional 4 h transport (shrink 2), using the following formula: shrink = (1 - (BW after transport / BW before transport)) ×100.

#### Feed intake and feeding behaviour

Pen intakes were determined by feed refusals recorded daily during the first week after transportation and weekly thereafter until d 28. Feed samples were collected on feed refusal days to determine feed dry matter (DM).

Eight pens (1 pen per treatment) were equipped with the GrowSafe feed bunk monitoring system (GrowSafe Systems, Airdrie, AB, Canada). Calves were fitted with radio frequency eat tags (RFID, Allflex Livestock Intelligence, St-Hyacinthe, QC, Canada) and each pen was equipped with two tubs which recorded individual feed intake during the study period. Feeding data was used to calculate meal size (kg/meal), meal duration (min/meal), meal frequency (min/meal), dry matter intake (kg/day), feeding duration (min/day) and feeding rate (g/min). A meal criterion of 300 s was selected based on previous studies in cattle [16,17].

### Behavioural assessments

#### Standing and lying

Standing and lying behaviour of a subset of 12 calves/treatment was recorded with accelerometers (Hobo pendant G, Onset Computer Corporation, Bourne, MA, USA) attached to the right hind leg of the calves using Vet Wrap (Professional Preference, AB, Calgary, Canada). Accelerometers were placed on the calves hind right leg immediately prior to LO1 in a vertical position with the X-axis pointing up towards the back of the animal and set to record data at 1-min intervals. Data from the days when the accelerometers were placed (d 0) and removed (d 14) were excluded from the analysis due to incomplete data.

#### Calf attitude and gait score

All calves attitude and gait were assessed after UN1 and UN2. An experienced observer assessed calves after exiting the squeeze chute while walking down an alley outside of the handling facilities. Attitude was evaluated using a 4-point scale [18]: 0: normal, cattle are bright and alert, hold their head up and readily move away from the observer. 1: mild depression, cattle’s attitude is slightly depressed but responds quickly to the observer and appears normal. 2: moderate depression, cattle stand with head down, ears droop, abdomen has lack of fill and may appear floppy, cattle move away slowly from observer. 3: severe depression, cattle stand with head down and very reluctant to move, very noticeable gauntness of abdomen.

Gait score was evaluated using a 5-point scale [19]: 0: normal (animal walks normally, with no apparent lameness or change in gait). 1: mild (walks easily and readily, bears full weight on foot and limb but has an observable gait alteration, stands on all four limbs, line of backbone normal. 2: moderate (reluctant to walk and bear weight but does use the limb to ambulate, short weight-bearing phase of stride, rests the affected limb when standing, increased periods of recumbency. May see arching of backbone. 3: severe (reluctant to stand, refuses to walk without stimulus. Non-weight bearing on affected limb, “hops” over limb rather than bearing weight, does not use limb when standing and lies down most of the time, backbone arched with caudoventral tip to pelvis). 4: non-ambulatory (recumbent, unable to rise, euthanasia often indicated).

#### Flight speed

The velocity at which animals exited the chute was collected after LO1, UN1, LO2, UN2, 7 h, 2, 14 and 28 d sampling. The time it took an animal to travel a predetermined distance (2 m) was electronically recorded using two sets of light beams as previously described by Burrow et al. [20]. Flight speed was collected as an indicator of temperament.

#### Physiological assessments

Blood samples were collected from a subset of 12 calves/treatment through jugular venipuncture at LO1, UN1, LO2, UN2, 7 h, 2 and 28 d post transport. Blood samples were collected into 10-mL non-additive tubes (BD vacutainer; Becton Dickinson Co., Franklin Lakes, NJ, USA) and left at room temperature for 1 h prior to centrifugation for 15 min at 2.5 × *g* at 4°C. Serum was decanted and frozen at -80°C for further analysis.

Non-esterified fatty acids (NEFA) were collected as an indicator of fat mobilization due to feed deprivation. NEFA concentrations were quantified using a colorimetric assay (HR Series NEFA-HR (2), FUJIFILM Wako Pure Chemical Corporation, Osaka, Japan). Intra- and inter-assay CV’s were 5.5% and 6.6%, respectively. Lactate was measured as an indicator of muscle damage using a L-Lactate fluorescence assay (Cayman Chemical Company, Ann Arbor, MI, USA) to quantify L-lactate concentration in serum. The intra- and inter-assay CV’s were 4.6% and 11.9%, respectively. Creatine kinase (CK) concentrations were quantified as an indicator of muscle damage using a colorimetric assay (EnzyChrom^™^ Creatine Kinase Assay Kit, BioAssay Systems, Hayward, CA, USA). Intra- and inter-assay CV’s were 5.5% and 2.8%, respectively. Haptoglobin was collected as in indicator of stress, inflammation, infection and trauma. Haptoglobin concentrations were quantified using a colorimetric assay (Tridelta Development Ltd., Maynooth, Co, Kildare, Ireland). The intra- and inter-assay CV’s were 7.7% and 5.1%, respectively. Complete blood cell count (CBC) was measured as an indicator of immune function using a HemaTrueHematology Analyzer (Heska, Loveland,Co). Serum cortisol concentrations were collected as an indicator of acute stress and concentrations were quantified using an immunoassay kit (DetectX Kit, Arbor Assays, Ann Arbor, MI, USA). Intra- and inter-assay CV’s were 6.8% and 7.9%, respectively. Hair was clipped from the forehead of subset of 12 animals/treatment at LO1 and d 28 to determine hair cortisol concentrations as an indicator of prolonged stress. Samples were stored at room temperature in plastic bags and analyzed as previously described by Moya et al. [21]. The amount of cortisol per unit of weight of powder hair was calculated using the formula described by Meyer et al. [22]. Cortisol concentrations were determined using a commercial kit (Salimetrics, State College, PA, USA). The intra- and inter-assay CV’s were 5.5% and 10.4%, respectively.

#### Morbidity and mortality

Morbidity and mortality were recorded for all calves over a 28 d experimental period.

### Statistical analysis

The normal distribution of the residuals was not assumed and therefore the models were “generalized” using PROC GLIMMIX (SAS, version 9.4, SAS Inst. Inc., Cary, NC). Although the normal distribution was not assumed, distributions from the exponential family (gamma, inverse Gaussian, log-normal, normal, exponential and shifted *t*) including the normal distribution, were tested and selected for each model based on the model fit statistics, i.e., Bayesian information criterion (BIC). After selecting the distribution, covariance structures: compound symmetry (CS), heterogeneous compound symmetry (CSH), variance component structure (VC), first-order autoregressive (AR1) and heterogeneous first-order autoregressive (ARH1) were tested and selected based on the BIC (S1 Table). The models were “mixed” due to the inclusion of fixed effects: transport and time (nested in rest) and random effects: animal and pen. Time was nested in rest to account for the missing sampling point (LO2) of the 0 h rest group. Covariates included in the model varied depending upon the variable assessed. Group, time of day, and flight speed were included as covariates in the analysis of physiological parameters (NEFA, lactate, haptoglobin, hair cortisol, serum cortisol, CK and CBC). Group and flight speed were included as covariates for the analysis of accelerometer data which was recorded daily. Group was included as a covariate for the analysis of parameters collected for all 320 animals (BW, feed refusals, ADG, rectal temperature and shrink). Data from 10 animals for shrink 1 and from 50 animals for shrink 2 were excluded from the analysis because the weight after transport was greater than the weight prior to transport. Feeding information from the week prior to the start of the trial was used as a covariate for GrowSafe feeding behaviour. GrowSafe data collected on d 0 was adjusted to the proportion of time animals were in the pen, as this varied between treatments. Area under the curve (AUC) was analysed using Graph Pad Prism v8 (GraphPad Prism, GraphPad Software, Inc, San Diego, CA, USA) for NEFA, lactate, haptoglobin, CK, hair and serum cortisol. Results are reported as least squares-means (*μ*) including the upper (u) and lower (l) limits at a 95% confidence. SAS PROC GLIMMIX iterated 1000 times at multiple levels of iterations (MAXOPT = 1000; NLOPTIONS MAXITER = 1000). A Bonferroni multiple comparisons correction was used. Scheffe-adjusted grouping was used for the presentation of least-squares means in all tables, while Bonferroni-adjusted *p*-values were reported in the text regarding the comparisons that were undertaken to address the research hypotheses. Reported differences were limited to comparisons of interest, such as differences between rest groups (R0, R4, R8, and R12), transport duration (12 h and 36 h), and their interactions. When there was a transport × time (nested in rest) interaction, comparisons of interest included either the same transport duration (e.g. 12-R0 vs 12-R8) or the same rest stop duration (e.g. 12-R0 vs 36-R0) at a particular sampling point. Statistical significance was *p* ≤ 0.05. In some cases, the *F*-test indicated the statistical significance of an interaction but there was no statistically significant difference between the levels of the interaction after Bonferroni’s adjustment for multiple comparisons. These interactions were not discussed.

## Results and discussion

### Body weight and shrink

**Fig 1:**
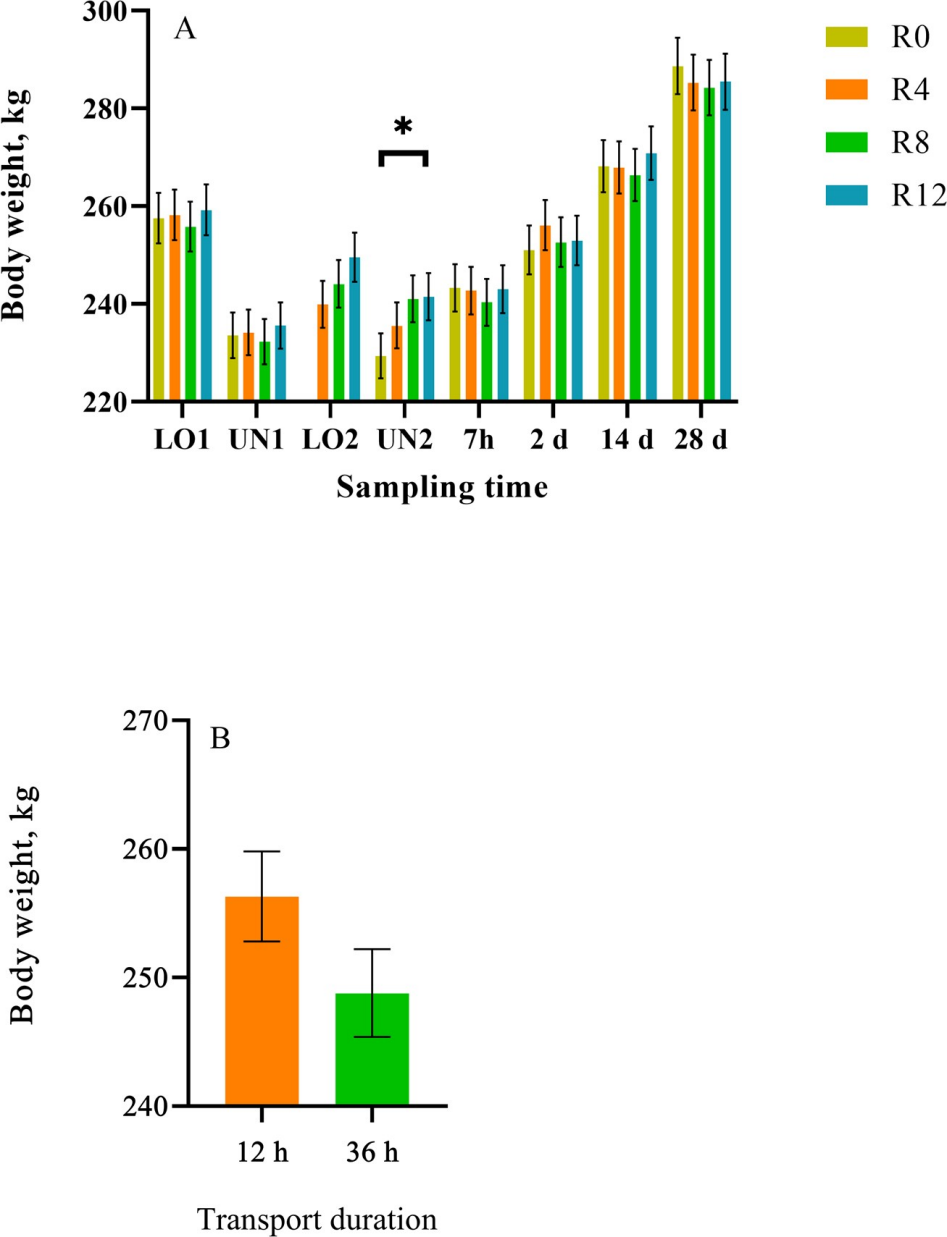
Least square means (± upper and lower limits) of body weight of conditioned black Angus and black Simmental calves. Body weight (kg) of calves receiving 0, 4, 8 and 12 h of rest and (B) body weight (kg) of calves transported for 12 and 36 h. ^a-b^Least square means with differing superscripts differ (*p* ≤ 0.05). *p ≤ 0.05.

**Fig 2:**
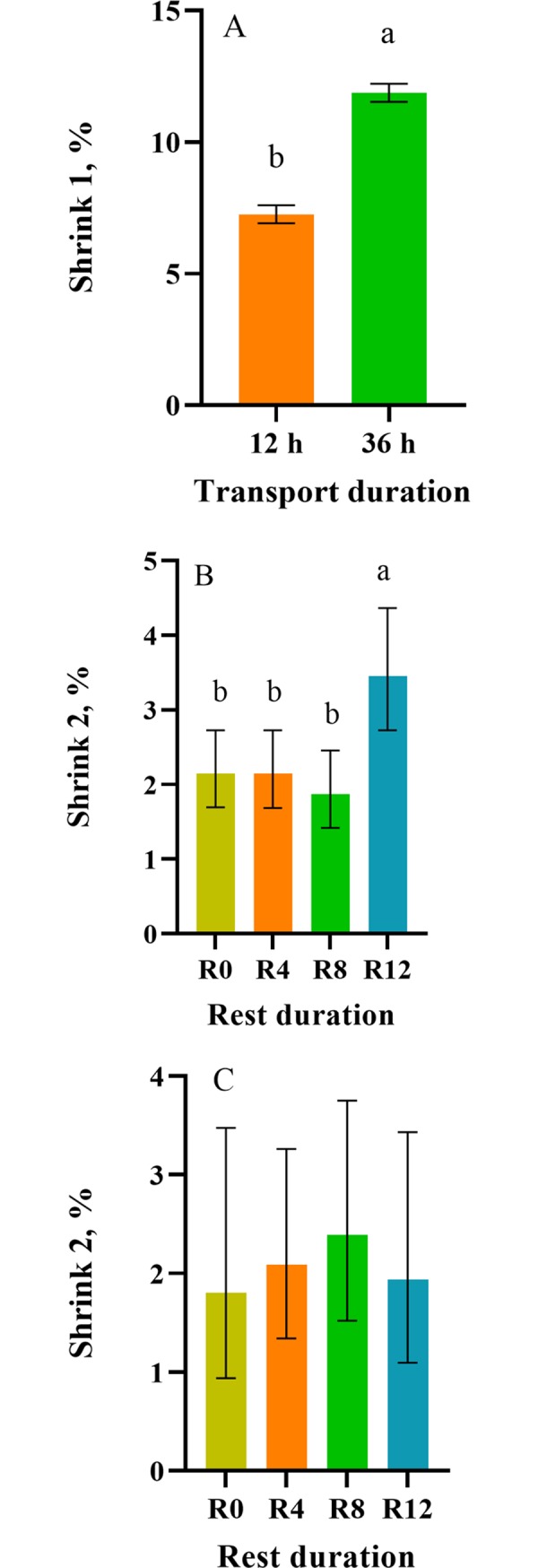
Least square means (± upper and lower limits) of shrink % of conditioned black Angus and black Simmental calves. Shrink % of calves after the initial 12 and 36 h transportation, (B) shrink % of calves receiving 0, 4, 8, and 12 h rest after the additional 4 h transport and (C) shrink % of a subset of 10 calves/treatment receiving 0, 4, 8, and 12 h rest after the additional 4 h transport taking feed intake into account. ^a-b^Least square means with differing superscripts differ (*p* ≤ 0.05).

A time (nested in rest) effect was observed for BW, where the R8 and R12 group had greater (*p* < 0.03) mean BW than the R0 group at UN2 (Fig 1A) (S2 and S4 Tables). These results were expected as the R8 and R12 group were provided access to feed and water prior to the 4 h transport, while the R0 group did not. Surprisingly, no differences were observed in mean BW between R0 and R4 calves. Treatment differences in weight were transient, as no differences were observed between rest stop groups 7 h after transportation which suggests that differences observed in BW at UN2 were likely due to gut fill. A transport effect (*p* = 0.04; *p* < 0.01) was observed for mean BW and shrink, where the 12 h-transported group had greater (*p* < 0.01) mean BW than the 36 h-transported group (Fig 1B) and the 36 h-transported group had greater (*p* < 0.01) mean shrink than the 12 h-transported group after the initial 12 and 36 h transport (Fig 2A). Shrink is defined as the loss of BW following periods of food and water deprivation due to the loss of urine and feces and body tissue, which can take from a few hours up to 30 d to recover [23]. Shrink rate averages 1%/h during the initial 3–4 h, and decreases to as low as 0.1% after 10 h or more on the truck [23]. Differences observed between 12 and 36 h-transported calves were expected, as animals that are transported for longer periods of time have greater weight loss (shrink) associated with urination, defecation and dehydration.

A rest effect (*p* < 0.01) was observed for shrink after the second 4 h transport, where the R12 group had greater (*p* ≤ 0.03) mean shrink than the R0, R4, and R8 group (Fig 2B). The greater mean shrink percentage observed in the R12 group was likely due to the rest stop duration, which allowed the animals the opportunity to eat and drink for a longer period of time. To account for feed intake during the rest period, shrink data from animals (10 calves/ treatment) housed in GrowSafe pens were analyzed using feed intake as a covariate. A transport effect (*p* < 0.01) was observed for transport duration, where the 36 h-transported group had greater mean shrink than the12 h-transported group, as observed in the previous results. However, no rest effects (*p* = 0.79) were observed for mean shrink after the 4 h transport (Fig 2C), suggesting that the shrink observed after the 4 h transport in the R12 group was mainly due to loss of gut fill. Caution should be taken when interpreting these results because the analysis using feed intake as a covariate was from a subset of 1 pen per treatment, therefore there was no treatment replication. Water consumption was not assessed in this experiment but ideally both feed and water intake should be taken into account when assessing the effect of rest stop duration on shrink.

Regarding shrink, Marti et al. [24] reported similar results, where no differences were observed for shrink in non-preconditioned calves (260 kg BW) transported for 15 h and given either no rest (4.0%), 5 (4.0%), 10 (4.4%) or 12 (4.4%) h of rest prior to an additional 5 h of transport. Nevertheless, Cooke et al. [25] reported greater shrink in preconditioned calves (229 kg BW) that were transported for 1290 km and were not rested (10.1%) compared to preconditioned calves that rested 2 h per 430 km (5.8%). Differences between studies could be due to the methods of calculation of shrink. That is, Marti et al. [24] calculated shrink during the additional 5 h transport after resting time, but Cooke et al. [25] calculated shrink over the entire journey, including the rest stops. Other differences between studies include transportation times prior to rest, rest stop durations, and types of cattle (newly weaned vs preconditioned). Inconsistencies in shrink data have been reported for preconditioned and non-preconditioned calves [26], greater shrink was observed in un-weaned calves compared to weaned calves [27], and studies reported that weaning closer to the time of transport resulted in greater shrink [28,29]. In the present study, the lack of a difference in shrink between rest stop treatment groups could be due to the fact that calves were conditioned prior to transportation. Other factors that can affect shrink include animal source (auction market vs ranch), time of loading, driver experience, time on truck, ambient temperature, and type of cattle [30].

### ADG

**Fig 3:**
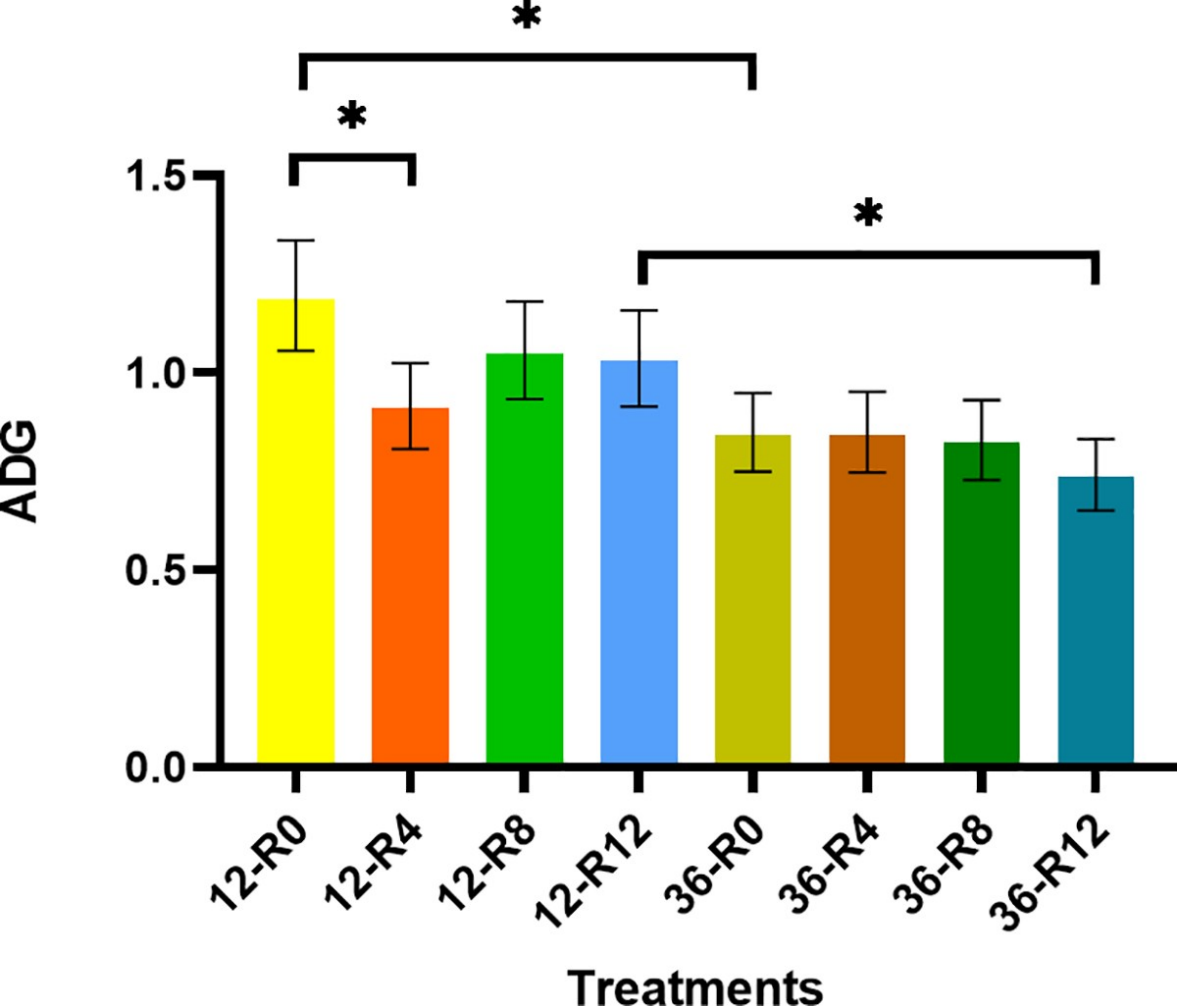
Least square means (± upper and lower limits) of ADG of conditioned black Angus and black Simmental calves during 28 days after transportation. *p ≤ 0.05.

A rest × transport interaction (*p* = 0.01) was observed for mean ADG where the 12-R0 group had greater mean ADG than the 12-R4 group and the 36-R0 group, while the 12-R12 group had greater mean ADG than the 36-R12 group (Fig 3). Overall, transport duration had an effect on ADG, where the 36 h-transported group had lower ADG than the 12 h-transported group with the exception of the 4 and 8 h rest groups. Interestingly, the 12-R0 group had greater mean ADG than the 12-R4 group, suggesting that a 4 h rest period was detrimental to ADG in comparison with calves that received no rest after a 12 hour transport. Regarding preconditioned calves, Cooke et al. [25] did not report a difference in ADG during the first 29 d after transport for calves that were transported continuously versus those that were rested. Regarding non-preconditioned calves, Marti el al. [24] observed greater ADG during the initial 25 d after transport from unrested calves and 5 h rested calves, in comparison to calves that received 10 and 15 h of rest after a 15 h transport and an additional 5 h of transport after rest. The authors suggested that longer journeys (including the resting time) could potentially affect ADG after transport. The lack of any statistical differences between the expected values of ADG of rest stop groups is hypothezised to be due to conditioning prior to transport. Further experimentation will be necessary to determine wither or not conditioning affects ADG.

### Feeding behaviour

Transport × time (nested in rest) interactions (*p* < 0.05) were observed for all feeding behaviour variables including DMI, meal size, meal duration, meal frequency, feeding rate, feeding intake and feeding time (S1 Fig). Mean DMI was greater (*p* < 0.05) in the 12-R0 group than the 36-R0 group on d 1 after transportation. Mean meal size and mean meal duration were greater (*p* < 0.05) in the 12-R8 group than the 12-R0 group on d 0 and 7, while the 12-R8 group had greater (*p* < 0.05) mean feeding intake than the 12-R4 and 36-R8 group, and greater (*p* < 0.05) mean feeding time than the 12-R4 group on d 0. The 12-R8 group had lower (*p* < 0.05) mean feeding time than the 36-R8 group on d 0, lower (*p* < 0.05) mean meal frequency than the 12-R0 group and greater (*p* < 0.05) mean meal duration than the 12-R0 group on d 7. The 36-R0 group had lower (*p* < 0.05) mean meal duration than the 12-R0 group on d 0, lower (*p* < 0.05) mean meal size than the 36-R8 group on d 27, greater (*p* < 0.05) mean meal frequency than the 36-R4 group on d 5, and both the 36-R0 and 36-R12 group had lower (*p* < 0.05) mean meal duration than the 36-R4 group on d 5. The 12-R0 group had greater (*p* < 0.05) mean feeding rate than the12-R8 group on d 0.

Overall, the 12-R0 group had greater DMI (d1) and meal duration (d0) than the 36-R0 group, while the 12-R8 group had greater feed intake (d0) but lower feeding time (d0), than the 36-R8 group. Calves that were rested for longer times were characterized by greater meal size, meal duration, feeding rate, feed intake and feeding time, and lower meal frequency, which indicated that rest affected feed consumption. This was attributed in part to a greater opportunity to access food during long rest periods, while short rest stops may result in more competition for access to the feeder, resulting in more frequent smaller meals and less intake. The effect of rest on feed consumption was attributed in part to fatigue among calves that were rested briefly, where animals prefer to rest rather than visit or compete for a space at the feed bunk. Differences in feeding behaviour varied between resting groups (0, 4, 8 and 12) and days (d 0, 1, 5, 7 and 27). In a previous study [24], calves that did not receive a rest stop spent less time at the feeder than calves that received 5, 10, or 15 h of rest after a 15 h transport and prior to an additional 5 h of transport. Marti et al. [24] hypothesized that fatigued calves would be motivated to lie down rather than eat, which could explain the differences observed between 12 and 36 h-transported groups in the present study, as well as the differences observed between rested groups. The current experiment, however, indicated that there were no differences in lengths of time spent standing and lying between transport groups on the days that differences were observed for feeding behaviour. Caution should be taken when interpreting feeding behaviour results (with the exception of DMI), as only 1 pen per treatment was equipped with Growsafe, and therefore there was no treatment replication.

### Standing and lying

A transport × time (nested in rest) interaction (*p* < 0.01) was observed for lying percentage where the 36-R8 group *(μ* = 75; u = 77.8%; l = 72.4%) had a greater (*p* < 0.01) lying percentage than the 12-R8 group (*μ* = 64; u = 67.4%; l = 61.0%) on d 1 after transportation. The greater amount of time spent lying by the 36 h-transported group was attributed to fatigue in comparison to the 12 h-transported group. This result was unexpected, as we hypothesized that 36 h-transported and R0 treatment groups would have greater mean lying duration due to fatigue, compared to 12 h-transported and R4, R8, and R12 calves. Marti et al. [24] reported greater standing duration in calves that received no rest, compared to animals that received 5, 10, or 15 h of rest after a 15 h transport and prior to an additional 5 h of transport, and speculated that this could be due to animals being unsettled. In the present study, no differences were observed in standing and lying behaviour in the 0 h-rest group but only in the 8 h-rest group. Reasons for varying results between studies could be due to differences in the way standing and lying data were summarized, as in the present study data was summarized by day while in the previous study, data was summarized by hour. Previous studies assessed standing, lying, feeding, and drinking behaviour during transportation [31,32,33] but, to the best of our knowledge, no studies have assessed the behaviour of conditioned calves continuously (per minute) during and after rest stops. In previous studies, calves could lie down during transportation, but in the present study the experimental calves were encouraged by the drivers to stand to reduce the potential for injury associated with standing calves stepping on other calves that were lying down in the trailer.

### Attitude and gait score

Subjective gait and attitude were assessed by an observer blind to the treatments after the 36 and 12 h transport, and after the additional 4 h transport to determine whether or not calves exhibited signs of lameness due to injury during loading and unloading, or fatigue. Many of the experimental calves had an attitude score of 0 at UN1 and UN2, 2 calves at UN1 and 14 calves at UN2 had attitude scores of 2. Many of the experimental calves had a gait score of 0, 2 calves had a gait score of 2 at UN1 and UN2. Therefore the attitude and gait scores were not analyzed due to a lack of variability. The scores indicated that many of the experimental calves were alert and were not lame. Low scores were attributed to the low-stress handling by trained personnel at the time of sampling and loading/unloading. However, low scores could also be due to cattle being ‘stoic’ animals, meaning that they do not display evident pain behaviour. Loading and unloading of cattle was hypothesized to be the most stressing events of transportation [34]. Individual gait and attitude assessments were done when animals excited the chute after UN1 and UN2 sampling. Individuals should be assessed at loading and unloading, however it was not possible to identify and assess calves at loading and unloading, due to the brevity of these events.

### NEFA

**Fig 4:**
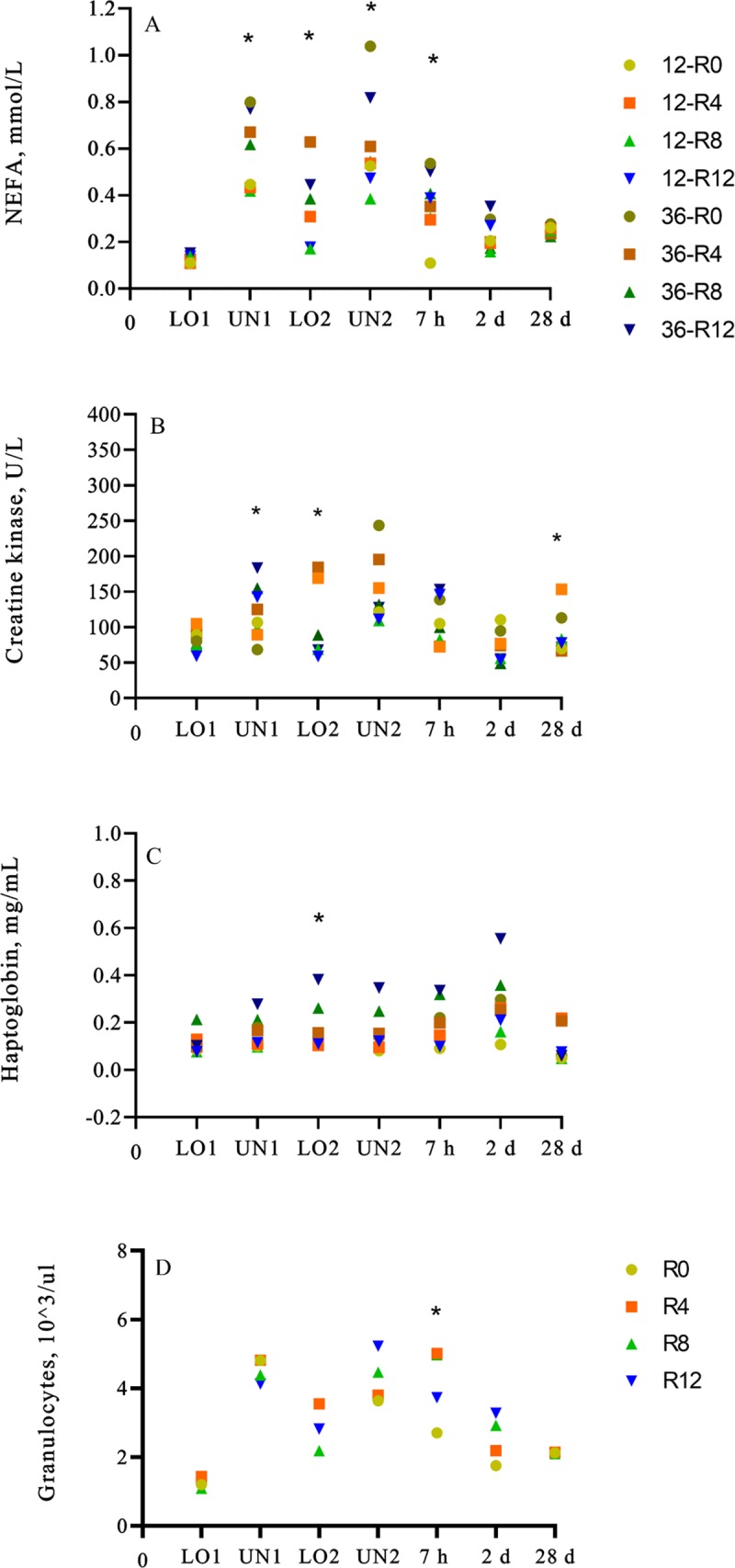
Least square means of physiological parameters of conditioned black Angus and black Simmental calves. (A) NEFA, (B) CK, (C) haptoglobin, and (D) granulocyte count of calves transported for 12 or 36 h and rested for 0, 4, 8 or 12 h. *p ≤ 0.05.

A transport × time (nested in rest) interaction (*p* < 0.01) was observed for mean NEFA, where the 36-R0 group had greater (*p* = 0.01) mean NEFA concentrations than 12-R0 group and, 36-R12 group had greater (*p* = 0.02) mean NEFA concentrations than 12-R12 group at UN1 (Fig 4A) (S3 and S5 Tables). At LO2, mean NEFA concentrations were greater (*p* < 0.01) in the 36-R4 group than the 12-R4 group and, the 36-R12 group had greater (*p* = 0.04) mean NEFA concentrations than the 12-R12 group. At UN2, mean NEFA concentrations were greater (*p* < 0.01) in the 36-R0 group than the 12-R0 group, while greater (*p* < 0.01) mean NEFA concentrations were observed in the 36-R12 group than the 12-R12 group. In addition, the 36-R0 group had greater (*p* < 0.01) mean NEFA concentrations than the 36-R4 and 36-R8 group at UN2. At 7 h, the 36-R0 group had greater (*P* < 0.01) mean NEFA concentrations than the 12-R0 group. Mean AUC for NEFA concentrations were greater (*p* < 0.01) in the 36-R0 group than the 12-R0, 36-R4 and 36-R8 group and greater (*p* < 0.01) in the 36-R12 group than the 12-R12 group. Overall, greater mean NEFA concentrations were observed in 36 h-transported calves compared to 12 h-transported calves, and from the R0 calves compared to the R4 and R8 calves from the 36 h-transported group.

Increased plasma NEFA concentrations suggest an increase in fatty acid mobilization due to energy deficiency caused by food restriction [35]. The 36 h-transported calves and R0 calves were food deprived for a longer period of time and therefore increased plasma NEFA was expected for these groups. Similar results were reported in cattle, where increased levels of plasma free fatty acids were correlated with time of food and water deprivation [36]. However, a different study reported that transport duration had no effect on NEFA concentrations in cattle [37]. Similar to the effect of transport duration, the effect of rest stop on NEFA concentrations is also inconsistent in the literature. For example, Cooke et al. [25], reported reduced NEFA concentrations on d 1 after 1290 km of transport in cattle rested for 2 h every 430 km compared to cattle that were not rested. However, this was contrary to findings by Marti et al. [24] who reported greater NEFA concentrations in non-conditioned calves that were transported for 15 h and received 10 h of rest compared to calves that received no rest or 15 h of rest prior to an additional 5 h of transport. In the present study of conditioned calves, 4 and 8 h-rest reduced NEFA concentrations compared to 0 h-rest calves after a 36 h-transport, however no rest stop effects were observed for calves transported for 12 h.

### Creatine kinase

A transport × time (nested in rest) interaction (*p* < 0.01) was observed for CK, where the 36-R12 group had greater (*p* = 0.02) mean CK concentrations than the 36-R0 group at UN1 (Fig 4B) (S6 Table). At LO2, greater (*p* = 0.02) mean CK concentrations were observed in the 12-R4 group than the 12-R8 calves, while greater (*p* = 0.02) mean CK concentrations were observed in the 12-R4 group than the 12-R12, the 12-R0 and 36-R4 group on d 28. Concentrations of CK increase in cases of muscle damage and muscle fatigue [38,39]. Although increases in CK concentrations have been reported in transported animals [38,39], no differences were observed in CK concentrations between calves that were standing or lying during transportation [31]. Contrary to our results, a previous study reported a direct relationship between transport duration and CK activity [32,40] as muscle exertion is required to maintain balance during transportation [41]. Based on the previous findings, Earley et al. [33] used CK concentrations and lying, as indicators of fatigue in transported and un-transported cattle. Their un-transported calves had a greater lying percentage than transported cattle 9 h after transportation, but no differences were observed between the mean CK concentrations of the treatment groups. Regarding CK concentrations, the results of the present study are contrary to what we expected, but are in accordance with other physiological parameters where no clear differences were observed between R0 calves and R4, R8 and R12 calves.

### Cortisol

No transport or rest effects (*p* > 0.10) were observed for hair and serum cortisol. These results may suggest that transportation did not generate a stress response, however, several studies have reported an increase in plasma cortisol after cattle transportation, suggesting transportation is indeed a stressful event [2, 5]. Lack of differences between treatments may be due to inadequate sampling times, as the cortisol peak likely occurred during transportation. For example, studies have reported peak plasma concentrations at 4.5 h during a 9 h transport [42], and at 12 h during a 31 h transport [31]. Cook et al. [43] suggested that cortisol levels decrease to pre-transport values 8–10 h after transportation. In the present study, samples were only collected before and after the 12 or 36 h transport, not during transport.

Contrary to our results, Cooke et al. [25] reported greater serum cortisol 1 d after 1290 km of transport without rest, compared to rested cattle, while Marti et al. [24], reported greater salivary cortisol concentrations in calves that did not receive a rest and animals that received 15 h of rest, compared to animals that received 5 and 10 h of rest after a 20 h transport Differences between our study and Marti et al. [24] could be attributed to calves being conditioned in the present study, which could have reduced the amount of stress experienced at the time of transportation. However, Cooke et al. [25] observed differences in rested and non rested preconditioned calves. Differences observed between studies are likely due to: differences in transport duration prior to a rest stop; rest stop duration; and sampling times.

### Haptoglobin

A transport × time (rest) interaction (*p* < 0.01) was observed for haptoglobin concentrations, where greater (*p* = 0.02) mean haptoglobin concentrations were observed in the 36-R12 group than the 12-R12 group at LO2 (Fig 4C) (S7 Table). The mean AUC for haptoglobin was greater (*p* = 0.05) in the 36-R12 than 12-R12 group. Cooke et al. [25] reported lower haptoglobin concentrations from cattle that received rest compared to cattle transported without rest. Marti et al. [24] reported no difference in haptoglobin concentrations between calves that were not rested and calves that were rested. Differences between studies are likely due to differences in transport and rest duration as well as sampling points. Corticosteroids can stimulate the production of acute phase proteins (haptoglobin) directly or indirectly through the activation IL-1 and IL-6 [44]. However, no treatment differences in cortisol concentrations were observed in the present study to support this. As mentioned previously, this could be due to a lack of sampling during transportation when cortisol concentrations have been reported to peak. Acute phase proteins (APP) increase in cases of infection, inflammation, and trauma [45]. The APP’s concentration in serum peak 24 to 48 h after the stimuli [45], however no differences were observed in haptoglobin concentrations on d 2 after transportation. Studies have shown an increase in APP after stressful situations in cattle such as transportation [46] and physical stress [47], however changes are commonly seen in serum amyloid-A or fibrinogen but not for haptoglobin [47,48]. These results suggest that the 36 h-transported calves had greater infection, inflammation or trauma than the 12 h-transported calves after a 12 h rest. Trauma during transport is more likely to occur during longer transport events associated with increased opportunity for injury and fatigue. Animals that are fatigued are more likely to lay down, which increases the risk for injury. This may explain the differences observed in haptoglobin concentrations for the 36-R12 compared to the 12-R12 group. However, if true, we would expect to see a difference between 12 and 36 h calves across rest groups, which was not observed. Another possible explanation is that animals in the 36-R12 group had subclinical infections that increased haptoglobin concentrations. Four out of the eight animals treated in the study were focal animals, two of them were in the 36-R12 group, one treated for foot rot and the other for fever, while 2 animals from the 12-R12 group were treated due to fever. These findings do not explain the differences observed for haptoglobin concentrations.

### CBC

A time (nested in rest) effect (*p* < 0.01) was observed for granulocytes, where the R4 group had greater (*p* < 0.01) mean granulocyte counts than the R0 group 7 h after transport (Fig 4D) (S8 Table), however, the observed values were within the normal range (1.3–7.5 × 10^3^/μl) [49]. Transport was reported previously to affect the microbiota of the nasopharynx, which can increase the susceptibility of calves to BRD [6]. Stress caused by weaning and transportation was associated with BRD, and an increase in corticosteroid release can lead to reduced immunity [5]. Transported animals can exhibit a classic stress response characterized by increased leucocytes, neutrophils, basophils, packed cell volume, and hemoglobin; and decreased lymphocytes, eosinophils, and monocytes [5]. Transportation changed the expression of neutrophil and anti-bacterial genes, which can contribute to excessive inflammation and tissue destruction, which can in turn contribute to the pathogenesis of infectious disease [42]. Calves transported immediately after weaning had higher cortisol levels than calves that were kept in the feedlot for two weeks prior to transport [50]. None of the other CBC values were affected (*p* < 0.10) by the rest stop durations and values were within the normal range. The reduced expression of indicators of stress at the time of transport and lack of differences between treatments were unexpected results. Lack of differences in CBC could be due to inadequate sampling times, high individual variability, or that in fact, immunity was unaffected by rest stop and transport duration. This is contrary to the studies previously mentioned. Weaning stress in combination with transport, may generate a stress response that is robust enough to affect the calves immune system, while transportation alone, may not be a stressor capable of affecting the immune system. In addition, transportation may have been less stressful for the calves in the present study because they had been previously transported from their ranch of origin to the research center. Based on the CBC results, conditioned calves were able to cope with the stress caused by transport.

Hematocrit (HCT), also known as packed cell volume (PCV), is the percentage of the blood volume occupied by red blood cells in the blood. Studies have used HCT to assess dehydration in animals transported for long distances [33,51]. Increased PCV has been reported after 24 h of transportation in steers [52]. In the present study, no differences (*p* < 0.10) were observed for hematocrit % between treatments, and surprisingly HCT values were within the normal range [49]. We expected greater HCT % in 36 h calves than 12 h calves, and in R0 calves compared to R4, R8 and R12 calves. Lack of differences in HCT % has been previously reported between food restricted calves for 8 h prior to an 8 h transport, in comparison to calves that had access to food prior to transport [51]. No signs of dehydration have also been reported in young calves transported for prolonged distances [53, 54, 55]. Lack of differences between transport and rest stop treatments in the present study, could be due to water absorption of the ruminal contents (30–40 L), which could prevent dehydration [56]. In addition, lack of differences between rest stop groups could be due to the short transport period (4 h) after the rest stop, which may have not been long enough for animals to show signs of dehydration after the rest period. However, this is unlikely as no differences were observed in HCT % after 12 and 36 h of transportation.

### Rectal temperature

A transport × time (rest) interaction (*p* < 0.01) was observed for rectal temperature, where the 36-R0 and 12-R4 group had greater (*p* < 0.05) mean rectal temperature than the 36-R4 group at UN1. At LO2, the 36-R12 group had greater (*p* < 0.05) mean rectal temperature than the 36-R4 group. At UN2, the 12-R4 and 12-R12 group had greater (*p* < 0.05) mean rectal temperature than the 12-R0 group, while the 36-R12 group had greater (*p* < 0.05) mean rectal temperature than the 36-R0 group. At 7h, the 12-R0 group had greater (*p* < 0.05) mean rectal temperature than the 12-R4 group. On d 2, the 36-R4 group had greater (*p* < 0.05) mean rectal temperature than the 36-R0 and the 36-R12 group, while the 12-R4 group had greater (*p* < 0.05) mean rectal temperature than the 12-R12 group. The differences in rectal temperature may lack biological significance as they were values from 0.1 to 0.7°C, which are small. The mean rectal temperature for all treatments at all sampling points was above the normal range (36.7–39.1°C) [57]. High rectal temperatures were observed in the present study, likely due to handling stress as animals were not sick prior to the start of the trial and the rectal temperatures observed at the initial sampling time point were numerically greater than the other sampling time points. Morbidity and mortality were 2.5% and 0%, respectively. Out of the eight sick animals in this study, one was treated for pinkeye, one for foot rot, and the rest were treated for fever.

Preconditioning has been shown to be an effective strategy for reducing BRD associated morbidity in feedlot calves [58, 59, 60]. Due to the fact that calves were weaned, transported, vaccinated, ear tagged, adapted to eat a grain diet from the feed bunk and adapted to drink from the water trough in the feedlot pens 18 to 26 days prior to transportation, they would have some benefit of partial preconditioning or “conditioning” [29]. The purpose of conditioning the animals was to separate the weaning and transport stress, ensuring that the effects being assessed in the current study were due to transport alone. Based on the current results, the calf management prior to transportation (conditioning) may have reduced the cumulative stress experienced by the calves such that any transport associated stress (long duration and lack of rest) did not overwhelm the coping mechanisms of the calves. Transport has been reported to be stressful for cattle [2,3], however the majority of transportation studies have been conducted in newly weaned calves, which is common industry practice [61], and it is possible that increased indicators of stress could be due to lack of conditioning instead of transportation. Grandin has referred to unvaccinated and newly weaned calves as unfit for transport [62]. Although rest stops could potentially improve animal welfare, they may also present a health risk due to exposure to other cattle which can carry different pathogens [63]. Calves between 200–300 kg suffered fewer post transport health issues, when transported for 32 h without a rest stop [62]. It is unclear whether or not this was due to the extending of transport stress and facilitating the exposure to novel pathogens, or inadequate rest stop conditions.

### Conclusion

Overall, 36 h-transportation resulted in lower mean BW, ADG, DMI intake and greater mean shrink %, lying percentage, haptoglobin, and NEFA concentrations than 12 h-transportation. With the exception of NEFA, rest stops did not have a consistent effect on welfare indicators contrary to what was expected. Future studies may assess whether or not newly weaned calves benefit from rest stops. Based on our results, conditioned calves can be said to benefit from shorter transportation, but rest stops did not reduce indicators of fatigue, dehydration, stress (haptoglobin and cortisol) or immune status.

## Supporting information

S1 Fig Feeding behaviour graphs. (DOCX)

S1 Dataset Physiological and behavioural raw data. (XLSX)

S1 Table Generalized linear mixed modelling (SAS POC GLIMMIX statements) indicating the response variable, the selected distribution, the link function, and the selected structure of the covariance matrix. (DOCX)

S2 Table Least square means (± upper and lower limits) of production and behavioural parameters of conditioned black Angus and black Simmental calves transported for 12 or 36 h and rested for 0, 4, 8 or 12 h^1^. (DOCX)

S3 Table Least square means (± upper and lower limits) of physiological parameters of conditioned black Angus and black Simmental calves transported for 12 or 36 h and rested for 0, 4, 8 or 12 h^1^. (DOCX)

S4 Table Least square means (± upper and lower limits) of BW (kg) of conditioned black Angus and black Simmental calves transported for 12 or 36 h and rested for 0, 4, 8 or 12 h^1^. (DOCX)

S5 Table Least square means (± upper and lower limits) of NEFA (mmol/L) concentrations of conditioned black Angus and black Simmental calves transported for 12 or 36 h and rested for 0, 4, 8 or 12 h^1^. (DOCX)

S6 Table Least square means (± upper and lower limits) of creatine kinase (U/L) concentrations of conditioned black Angus and black Simmental calves transported for 12 or 36 h and rested for 0, 4, 8 or 12 h^1^. (DOCX)

S7 Table Least square means (± upper and lower limits) of haptoglobin (mg/mL) concentrations of conditioned black Angus and black Simmental calves transported for 12 or 36 h and rested for 0, 4, 8 or 12 h^1^. (DOCX)

S8 Table Least square means (± upper and lower limits) of granulocyte cell count (10^3^/μl) of conditioned black Angus and black Simmental calves transported for 12 or 36 h and rested for 0, 4, 8 or 12 h^1^. (DOCX)
