# Supplementary material for: Effect of transport and rest stop duration on the welfare of conditioned cattle transported by road
Source: PLoS One. 2020 Mar 2;15(3):e0228492. doi: 10.1371/journal.pone.0228492 (PMC7051828; doi:10.1371/journal.pone.0228492)

S1 Fig. Least square means of feeding behaviour of conditioned black Angus and black Simmental calves.

1. DMI, (B) meal size, (C) meal duration, (D) meal frequency, (E) feeding rate, (F) feeding intake and (G) feeding time of calves transported for 12 or 36 h and rested for 0, 4, 8 or 12 h. ^*^*p* ≤ 0.05.


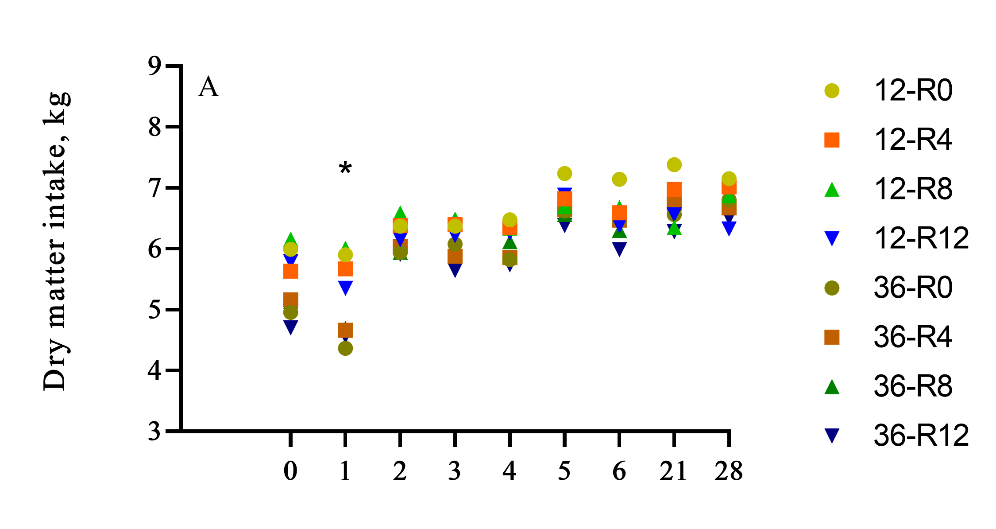


**
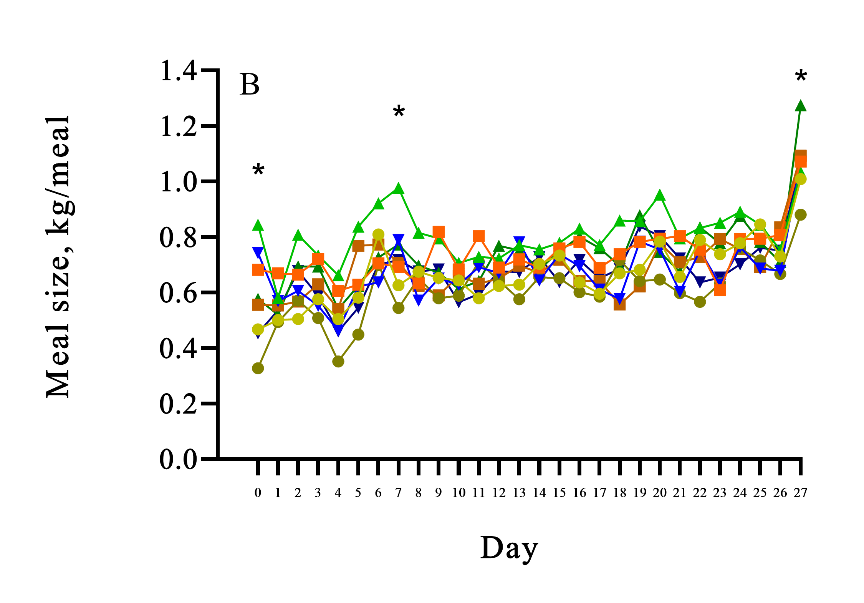
**

**
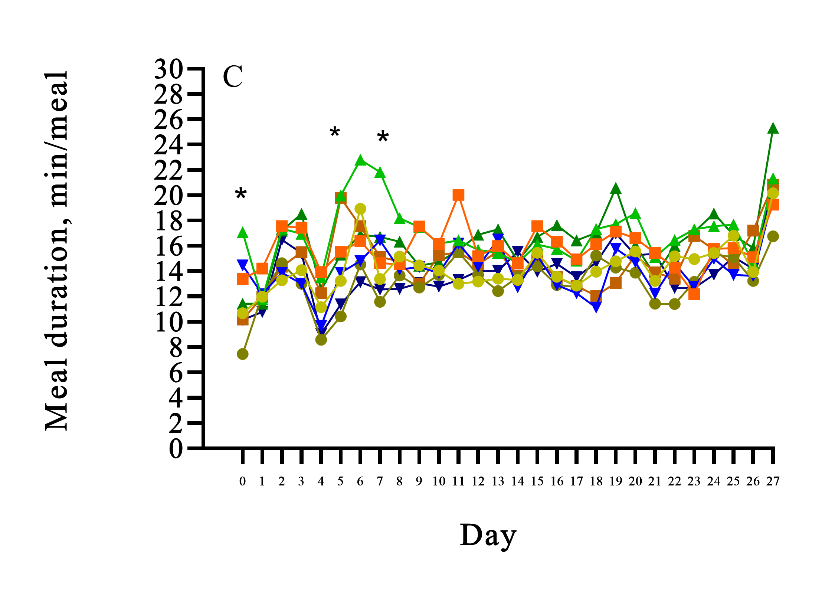
**


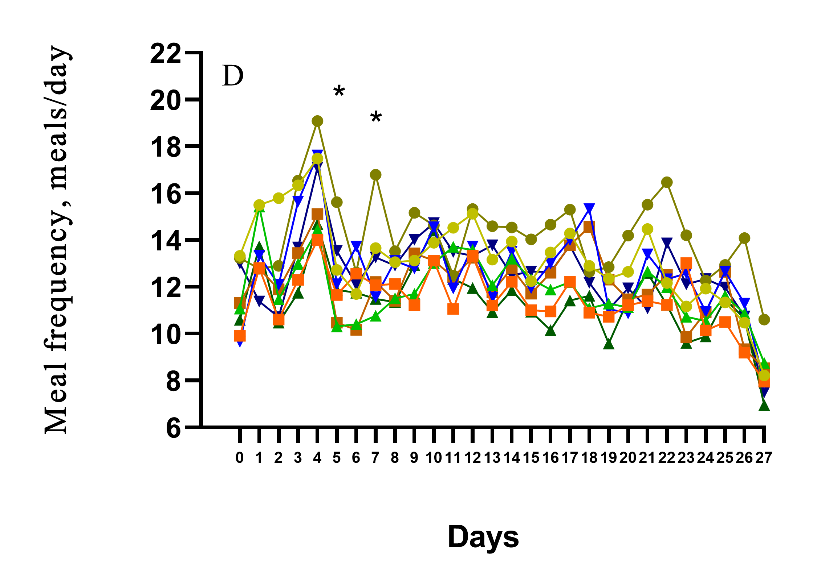


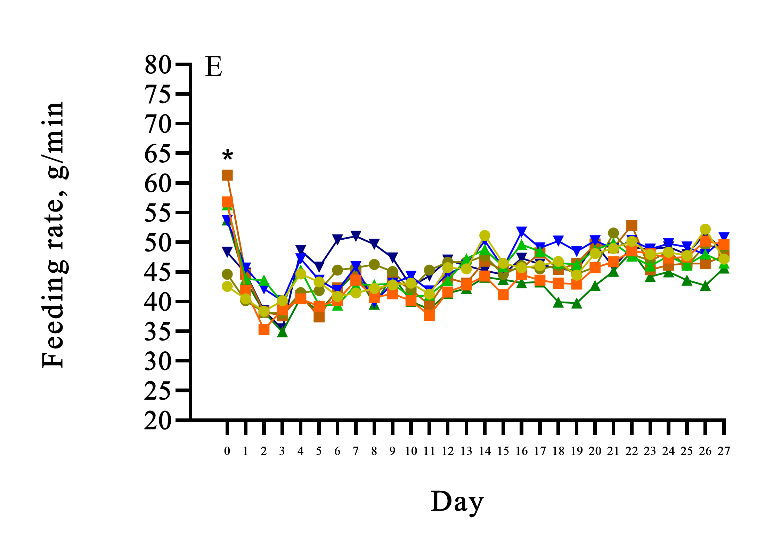


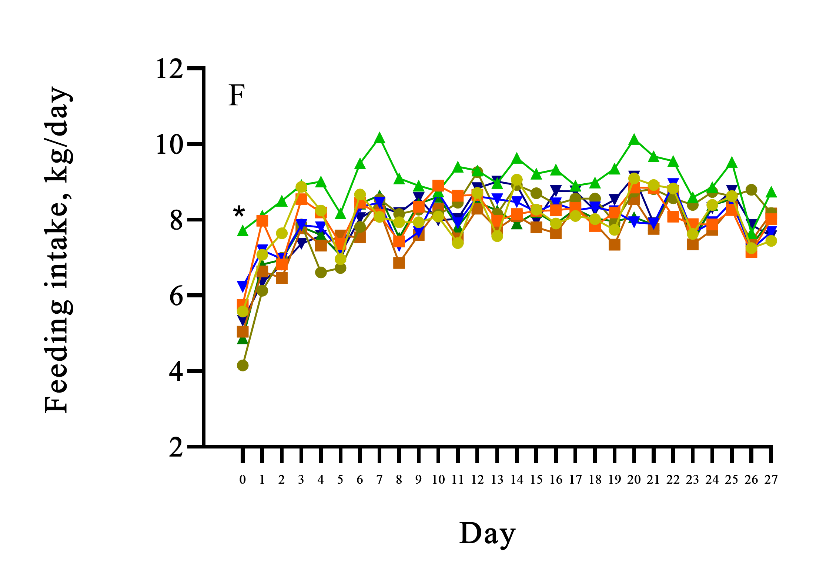


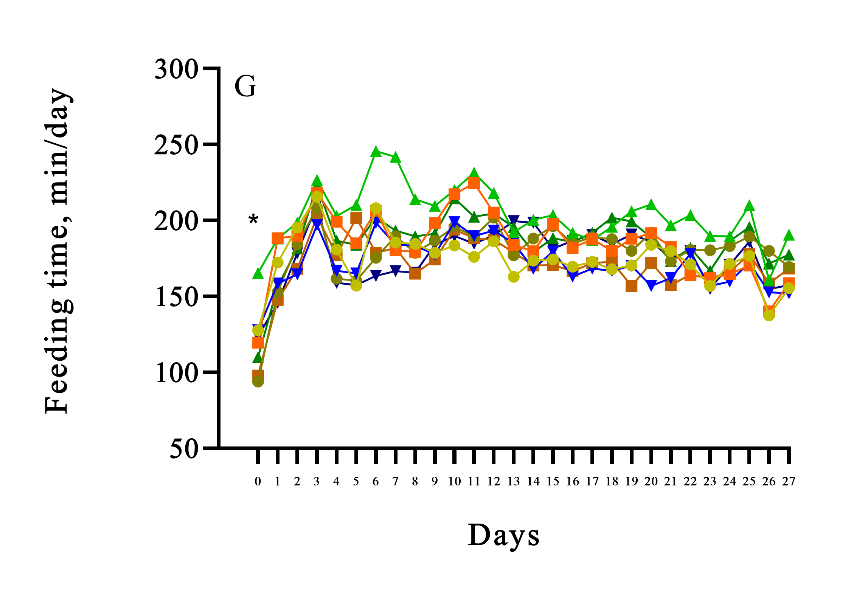

Supplement: S1 Fig — (DOCX) [file pone.0228492.s001.docx]
