# Supplementary material for: Effect of transport and rest stop duration on the welfare of conditioned cattle transported by road
Source: PLoS One. 2020 Mar 2;15(3):e0228492. doi: 10.1371/journal.pone.0228492 (PMC7051828; doi:10.1371/journal.pone.0228492)
Supplement: S1 Table — (DOCX) [file pone.0228492.s003.docx]

S1 Table. Generalized linear mixed modelling (SAS POC GLIMMIX statements) indicating the response variable, the selected distribution, the link function, and the selected structure of the covariance matrix.

| **Experimental variable** | **Distribution** | **Link function** | **Covariance structure** |
| --- | --- | --- | --- |
| NEFA | N | Identity | CSH |
| Haptoglobin | T | Identity | ARH(1) |
| Hair cortisol | N | Identity | CSH |
| Serum cortisol | GAMMA | Log | VC |
| Lactate | GAMMA | Log | VC |
| Creatine Kinase | GAMMA | Log | ARH(1) |
| Weight | LOGN | Identity | VC |
| ADG | GAMMA | Log | - |
| Shrink 1 | N | Identity | - |
| Shrink 2 | EXP | Log | - |
| Shrink 1 cov | LOGN | Identity | - |
| Shrink 2 cov | LOGN | Identity | - |
| Feeding Time | GAMMA | Log | VC |
| Feeding Intake | GAMMA | Log | VC |
| Feeding Rate | LOGN | Identity | VC |
| Meal frequency | GAMMA | Log | VC |
| Meal duration | LOGN | Identity | VC |
| Meal size | N | Identity | VC |
| Inter meal interval | LOGN | Log | VC |
| Flight Speed | LOGN | Identity | AR(1) |
| Rectal temperature | GAMMA | Log | CSH |
| Weighbacks | T | Identity | AR(1) |
| Standing duration | LOGN | Identity | ARH(1) |
| Lying duration | GAMMA | Log | CSH |
| Standing percentage | BETA | Logit | CSH |
| Lying percentage | BETA | Logit | AR(1) |
| RBC | IG | Reciprocal Square | ARH(1) |
| WBC | IG | Reciprocal Square | ARH(1) |
| Lymphocyte % | GAMMA | Log | CSH |
| Monocyte % | IG | Reciprocal Square | VC |
| Granulocyte % | LOGN | Identity | ARH(1) |
| Lymphocytes | LOGN | Identity | ARH(1) |
| Monocytes | N | Identity | ARH(1) |
| Granulocytes | N | Identity | ARH(1) |
| Platelets | GAMMA | Log | AR(1) |
| MCV | IG | Reciprocal Square | AR(1) |
| HCT | GAMMA | Log | AR(1) |
| MCH | LOGN | Identity | ARH(1) |
| MCHC | IG | Reciprocal Square | AR(1) |
| RDW | IG | Reciprocal Square | VC |
| RDWa | LOGN | Identity | CSH |
| MPV | LOGN | Identity | ARH(1) |
| HGB | GAMMA | Log | AR(1) |
