# Supplementary material for: Effect of transport and rest stop duration on the welfare of conditioned cattle transported by road
Source: PLoS One. 2020 Mar 2;15(3):e0228492. doi: 10.1371/journal.pone.0228492 (PMC7051828; doi:10.1371/journal.pone.0228492)
Supplement: S2 Table — (DOCX) [file pone.0228492.s004.docx]

S2 Table. Least square means (± upper and lower limits) of production and behavioural parameters of conditioned black Angus and black Simmental calves transported for 12 or 36 h and rested for 0, 4, 8 or 12 h^1^

|  | Treatment^2^ | | | | | | | |  |  | *P*-value | | | | |
| --- | --- | --- | --- | --- | --- | --- | --- | --- | --- | --- | --- | --- | --- | --- | --- |
| *Item* | 12-R0 | 12-R4 | 12-R8 | 12-R12 | 36-R0 | 36-R4 | 36-R8 | 36-R12 | Minimum | Maximum | Trans | Rest | Trans×Rest | Time(R) | Time(R)×Trans |
| Body weight, kg | 257 | 254 | 256 | 258 | 248 | 249 | 247 | 251 | 245.7 | 259.6 | <0.01 | 0.88 | 0.93 | <0.01 | <0.01 |
| Shrink 1, % | 7.4 | 7.6 | 7.0 | 7.1 | 11.9 | 11.2 | 11.8 | 12.5 | 8.88 | 10.26 | <0.01 | 0.52 | 0.09 | - | - |
| Shrink 2, % | 2.6 | 2.6 | 1.8 | 3.9 | 1.8 | 1.7 | 2.1 | 3.1 | 1.73 | 3.44 | 0.09 | 0.01 | 0.37 | - | - |
| Shrink 2 cov, % | 0.8 | 1.3 | 0.5 | 0.4 | 0.9 | 0.5 | 1.0 | 0.4 | 0.04 | 1.42 | 0.81 | 0.61 | 0.15 | - | - |
| ADG, | 1.2^a^ | 0.9^b^ | 1.0^a^ | 1.0^a^ | 0.8^b^ | 0.9^b^ | 0.8^b^ | 0.7^b^ | 0.83 | 0.87 | <0.01 | 0.14 | 0.01 | - | - |
| DMI, kg | 6.3 | 6.1 | 6.1 | 5.9 | 5.3 | 5.4 | 5.4 | 5.1 | 5.51 | 5.90 | <0.01 | 0.01 | 0.42 | <0.01 | <0.01 |
| Meal size, kg/meal | 0.7 | 0.7 | 0.7 | 0.7 | 0.6 | 0.7 | 0.7 | 0.7 | 0.59 | 0.81 | 0.61 | 0.58 | 0.95 | <0.01 | <0.01 |
| Meal duration, min/meal | 26.9 | 27.6 | 27.4 | 26.4 | 25.7 | 26.7 | 28.0 | 26.5 | 25.7 | 28.1 | 0.41 | 0.07 | 0.46 | <0.01 | <0.01 |
| Meal frequency meals/day | 13.0 | 11.5 | 12.3 | 12.4 | 13.9 | 11.8 | 11.3 | 12.4 | 11.3 | 13.5 | 0.92 | 0.01 | 0.43 | <0.01 | <0.01 |
| Feed intake, kg/day | 8.1 | 8.0 | 8.9 | 8.0 | 8.0 | 7.7 | 7.6 | 7.9 | 7.54 | 8.54 | 0.02 | 0.52 | 0.11 | <0.01 | <0.01 |
| Feeding rate, g/min | 38^ab^ | 38^ab^ | 38^ab^ | 39^a^ | 38^ab^ | 38^ab^ | 37^ab^ | 38^b^ | 37.3 | 38.8 | 0.58 | 0.36 | 0.03 | <0.01 | <0.01 |
| Feeding time, min/day | 182 | 181 | 195 | 171 | 177 | 168 | 188 | 175 | 167.1 | 193.5 | 0.24 | 0.03 | 0.63 | <0.01 | <0.01 |
| Inter-meal duration, s | 5915 | 6682 | 6358 | 6347 | 5519 | 6619 | 6135 | 6022 | 5666.4 | 6748.1 | 0.24 | 0.01 | 0.93 | <0.01 | <0.01 |
| Lying, % | 60 | 60 | 59 | 61 | 61 | 61 | 61 | 60 | 58.8 | 61.9 | 0.09 | 0.69 | 0.22 | <0.01 | <0.01 |
| Standing % | 48 | 40 | 37 | 32 | 58 | 52 | 50 | 25 | 27.8 | 59.2 | 0.30 | 0.04 | 0.65 | <0.01 | <0.01 |
| Lying bout, min | 69.6^ab^ | 68.8^ab^ | 61.0^b^ | 71.0^ab^ | 67.1^ab^ | 67.1^ab^ | 75.9^a^ | 66.4^ab^ | 62.4 | 74.9 | 0.50 | 1.00 | <0.01 | 0.01 | 0.14 |
| Standing bout, min | 45.8 | 47.0 | 45.3 | 46.2 | 45.8 | 46.9 | 50.6 | 45.9 | 41.6 | 54.4 | 0.55 | 0.87 | 0.70 | <0.01 | 0.43 |
| Flight speed, m/s | 1.9 | 2.2 | 1.8 | 2.0 | 2.2 | 2.0 | 2.1 | 2.1 | 1.68 | 2.48 | 0.41 | 0.88 | 0.51 | <0.01 | 0.96 |

Scheffe *P*-values are presented in the table, however, superscripts correspond to Bonferroni adjusted *P*-values for comparisons of interest. Least square means within a row with differing superscripts differ ( *P*  ≤ 0.05)

^1^Values in the table represent the mean of LO1 and d 28 for hair cortisol samples; the means of LO1, UN1, LO2, UN2, 7 h, 2 and 28 after UN2 for scrotal temperature (SCT), rectal temperature (Temp) and white blood cell count (WBC).

^2^ Transport: 12: 12 h of transportation and 36: 36 h of transportation. Rest stop: R0: 0 h of rest, R4: 4 h of rest, R8: 8 h of rest and R12: 12 h of rest.
