# Supplementary material for: Effect of transport and rest stop duration on the welfare of conditioned cattle transported by road
Source: PLoS One. 2020 Mar 2;15(3):e0228492. doi: 10.1371/journal.pone.0228492 (PMC7051828; doi:10.1371/journal.pone.0228492)
Supplement: S3 Table — (DOCX) [file pone.0228492.s005.docx]

S3 Table. Least square means (± upper and lower limits) of physiological parameters of conditioned black Angus and black Simmental calves transported for 12 or 36 h and rested for 0, 4, 8 or 12 h^1^

|  | Treatment^2^ | | | | | | | |  |  | *P*-value | | | | |
| --- | --- | --- | --- | --- | --- | --- | --- | --- | --- | --- | --- | --- | --- | --- | --- |
| *Item* | 12-R0 | 12-R4 | 12-R8 | 12-R12 | 36-R0 | 36-R4 | 36-R8 | 36-R12 | Minimum | Maximum | Trans | Rest | Trans*Rest | Time(R) | Time(R)×Trans |
| Hair cortisol, pg/mg | 8.4 | 6.4 | 5.7 | 5.5 | 5.7 | 8.6 | 4.4 | 5.3 | 3.76 | 8.73 | 0.57 | 0.14 | 0.24 | 0.99 | 0.84 |
| Cortisol, | 27.4 | 31.4 | 31.0 | 30.8 | 31.2 | 32.8 | 30.8 | 37.0 | 26.47 | 37.60 | 0.12 | 0.58 | 0.67 | <0.01 | 0.25 |
| Haptoglobin, mg/ml | 0.1 | 0.2 | 0.1 | 0.1 | 0.2 | 0.2 | 0.3 | 0.3 | 0.01 | 0.33 | 0.05 | 0.96 | 0.74 | <0.01 | <0.01 |
| L-lactate, mmol | 2.6 | 2.4 | 2.0 | 2.0 | 2.8 | 2.3 | 2.0 | 2.2 | 1.77 | 2.96 | 0.82 | 0.38 | 0.88 | <0.01 | 0.07 |
| NEFA, mmol/L | 0.28^c^ | 0.30^c^ | 0.26^d^ | 0.31^c^ | 0.51^a^ | 0.40^ac^ | 0.35^bc^ | 0.47^ab^ | 0.30 | 0.42 | <0.01 | 0.01 | 0.05 | <0.01 | <0.01 |
| Creatine kinase | 99.0 | 111.2 | 80.6 | 85.5 | 112.2 | 106.0 | 91.2 | 94.8 | 82.24 | 115.83 | 0.13 | 0.07 | 0.54 | <0.01 | <0.01 |
| Rectal temp, °C | 39.8 | 40.0 | 39.8 | 39.8 | 39.7 | 39.8 | 39.8 | 39.9 | 39.8 | 39.9 | 0.05 | 0.09 | 0.06 | <0.01 | <0.01 |
| HCT, % | 29.0 | 31.2 | 31.0 | 31.3 | 30.3 | 30.0 | 30.8 | 30.8 | 29.20 | 32.00 | 0.77 | 0.34 | 0.36 | <0.01 | <0.01 |
| WBC | 10.0 | 10.8 | 10.0 | 10.3 | 9.7 | 9.9 | 9.9 | 9.9 | 9.15 | 11.33 | 0.28 | 0.82 | 0.92 | <0.01 | 0.19 |
| Lymphocytes | 65.4 | 61.2 | 61.8 | 61.7 | 66.2 | 64.4 | 62.2 | 61.4 | 59.27 | 67.03 | 0.43 | 0.33 | 0.78 | <0.01 | 0.23 |

Scheffe *P*-values are presented in the table, however, superscripts correspond to Bonferroni adjusted *P*-values for comparisons of interest. Least square means within a row with differing superscripts differ ( *P*  ≤ 0.05).

^1^Values in the table represent the mean of LO1 and d 28 for hair cortisol; the means of LO1, UN1, LO2, UN2, 7 h, 2 and 28 d after UN2 for cortisol, haptoglobin, lactate scout, lactate kits, NEFA, creatine kinase, rectal temp, HCT, WBC and lymphocytes.

^2^ Transport: 12: 12 h of transportation and 36: 36 h of transportation. Rest stop: R0: 0 h of rest, R4: 4 h of rest, R8: 8 h of rest and R12: 12 h of rest.
