# Supplementary material for: Effect of transport and rest stop duration on the welfare of conditioned cattle transported by road
Source: PLoS One. 2020 Mar 2;15(3):e0228492. doi: 10.1371/journal.pone.0228492 (PMC7051828; doi:10.1371/journal.pone.0228492)
Supplement: S4 Table — (DOCX) [file pone.0228492.s006.docx]

S4 Table. Least square means (± upper and lower limits) of BW (kg) of conditioned black Angus and black Simmental calves transported for 12 or 36 h and rested for 0, 4, 8 or 12 h^1^

|  | Treatments^2^ | | | |  |  |  |
| --- | --- | --- | --- | --- | --- | --- | --- |
| *Item* | R0 | R4 | R8 | R12 | Maximum | Minimum | *P*-value |
| LO1 | 257.5 | 258.2 | 255.7 | 259.2 | 262.84 | 252.54 | 0.81 |
| UN1 | 233.5 | 234.1 | 232.2 | 235.5 | 238.57 | 229.22 | 0.80 |
| LO2 | - | 239.9 | 244.1 | 249.5 | 249.40 | 239.63 | 0.02 |
| UN2 | 229.3^b^ | 235.5^ab^ | 241.0^a^ | 241.4^a^ | 241.60 | 232.12 | <0.01 |
| 7 h | 243.2 | 242.7 | 240.3 | 243.0 | 247.16 | 237.48 | 0.82 |
| 2 d | 251.0 | 256.1 | 252.6 | 252.9 | 258.26 | 248.12 | 0.56 |
| 14 d | 268.1 | 267.8 | 266.3 | 270.8 | 273.67 | 262.94 | 0.70 |
| 28 d | 288.6 | 285.2 | 284.1 | 285.4 | 291.60 | 280.17 | 0.73 |

Scheffe *P*-values are presented in the table, however, superscripts correspond to Bonferroni adjusted *P*-values. ^ab^Least square means within a row with differing superscripts differ (*P*  ≤ 0.05).

^1^Values in the table represent the mean of BW for each treatment at LO1, UN1, LO2, UN2, 7 h, 2, 14 and 28 d.

^2^ Rest stop: R0: 0 h of rest, R4: 4 h of rest, R8: 8 h of rest and R12: 12 h of rest.
