# Supplementary material for: Effect of transport and rest stop duration on the welfare of conditioned cattle transported by road
Source: PLoS One. 2020 Mar 2;15(3):e0228492. doi: 10.1371/journal.pone.0228492 (PMC7051828; doi:10.1371/journal.pone.0228492)
Supplement: S5 Table — (DOCX) [file pone.0228492.s007.docx]

S5 Table. Least square means (± upper and lower limits) of NEFA (mmol/L) concentrations of conditioned black Angus and black Simmental calves transported for 12 or 36 h and rested for 0, 4, 8 or 12 h^1^

|  | Treatments^2^ | | | | | | | |  |  |  |
| --- | --- | --- | --- | --- | --- | --- | --- | --- | --- | --- | --- |
| *Item* | 12-R0 | 12-R4 | 12-R8 | 12-R12 | 36-R0 | 36-R4 | 36-R8 | 36-R12 | Maximum | Minimum | *P* -value |
| LO1 | 0.1 | 0.1 | 0.1 | 0.1 | 0.1 | 0.1 | 0.1 | 0.2 | 0.17 | 0.08 | 0.79 |
| UN1 | 0.4^b^ | 0.4 | 0.4 | 0.4^f^ | 0.8^a^ | 0.7 | 0.6 | 0.8^e^ | 0.71 | 0.44 | <0.01 |
| LO2 | - | 0.3^d^ | 0.2 | 0.2^f^ | - | 0.6^c^ | 0.4 | 0.4^e^ | 0.47 | 0.24 | <0.01 |
| UN2 | 0.5^b^ | 0.5 | 0.4 | 0.5^h^ | 1.0^aA^ | 0.6^B^ | 0.5^B^ | 0.8^gAB^ | 0.75 | 0.48 | <0.01 |
| 7 h | 0.1^b^ | 0.3 | 0.3 | 0.4 | 0.5^a^ | 0.4 | 0.4 | 0.5 | 0.46 | 0.26 | <0.01 |
| 2 d | 0.2 | 0.2 | 0.2 | 0.3 | 0.3 | 0.2 | 0.2 | 0.4 | 0.31 | 0.16 | 0.09 |
| 28 d | 0.3 | 0.3 | 0.2 | 0.3 | 0.3 | 0.2 | 0.2 | 0.2 | 0.31 | 0.19 | 0.87 |

Scheffe *P*-values are presented in the table, however, superscripts correspond to Bonferroni adjusted *P*-values for comparisons of interest. ^ab^ superscripts indicate differences between the R0 groups, ^cd^ between the R4 groups, and ^ef^ between the R12 groups. ^AB^ superscritps indicate differences of interest within the 36 h transport group. Least square means within a row with differing superscripts differ (*P* ≤ 0.05).

^1^Values in the table represent the mean of NEFA concentrations for each treatment at LO1, UN1, LO2, UN2, 7 h, 2 and 28 d.

^2^ Transport: 12: 12 h of transportation and 36: 36 h of transportation. Rest stop: R0: 0 h of rest, R4: 4 h of rest, R8: 8 h of rest and R12: 12 h of rest.
