# Supplementary material for: Effect of transport and rest stop duration on the welfare of conditioned cattle transported by road
Source: PLoS One. 2020 Mar 2;15(3):e0228492. doi: 10.1371/journal.pone.0228492 (PMC7051828; doi:10.1371/journal.pone.0228492)
Supplement: S6 Table — (DOCX) [file pone.0228492.s008.docx]

S6 Table. Least square means (± upper and lower limits) of creatine kinase (U/L) concentrations of conditioned black Angus and black Simmental calves transported for 12 or 36 h and rested for 0, 4, 8 or 12 h^1^

|  | Treatments^2^ | | | | | | | |  |  |  |
| --- | --- | --- | --- | --- | --- | --- | --- | --- | --- | --- | --- |
| *Item* | 12-R0 | 12-R4 | 12-R8 | 12-R12 | 36-R0 | 36-R4 | 36-R8 | 36-R12 | Minimum | Maximum | *P* -value |
| LO1 | 89.6 | 104.7 | 75.7 | 59.2 | 80.4 | 91.5 | 77.4 | 71.3 | 114.18 | 57.78 | 0.32 |
| UN1 | 106.8 | 89.6 | 98.6 | 142.4 | 68.5 | 125.3 | 155.2 | 183.6 | 176.82 | 83.15 | 0.01 |
| LO2 |  | 169.0 | 69.3 | 58.7 |  | 184.5 | 89.3 | 67.8 | 164.00 | 69.35 | 0.00 |
| UN2 | 121.6 | 155.1 | 109.5 | 111.2 | 243.6 | 195.7 | 132.1 | 127.6 | 228.08 | 99.83 | 0.00 |
| 7 h | 105.1 | 72.4 | 82.7 | 145.9 | 138.7 | 73.3 | 99.8 | 153.2 | 154.72 | 77.66 | 0.28 |
| 2 d | 110.5 | 77.0 | 56.2 | 53.4 | 95.0 | 74.2 | 49.3 | 55.3 | 114.14 | 45.09 | 0.83 |
| 28 d | 69.7 | 153.4 | 83.5 | 77.9 | 113.2 | 66.9 | 75.3 | 71.5 | 134.07 | 59.00 | 0.00 |

Scheffe *P*-values are presented in the table, however, superscripts correspond to Bonferroni adjusted *P*-values for comparisons of interest. ^ab^ superscripts indicate differences between the R4 group. ^AB^ superscripts indicate differences of interest within the 36 h transport group. ^CD^ superscripts indicate differences of interest within the 12 h transport group. Least square means within a row with differing superscripts differ (*P* ≤ 0.05).

^1^Values in the table represent the mean of creatine kinase concentrations for each treatment at LO1, UN1, LO2, UN2, 7 h, 2 and 28 d.

^2^ Transport: 12: 12 h of transportation and 36: 36 h of transportation. Rest stop: R0: 0 h of rest, R4: 4 h of rest, R8: 8 h of rest and R12: 12 h of rest.
