# Supplementary material for: Effect of transport and rest stop duration on the welfare of conditioned cattle transported by road
Source: PLoS One. 2020 Mar 2;15(3):e0228492. doi: 10.1371/journal.pone.0228492 (PMC7051828; doi:10.1371/journal.pone.0228492)
Supplement: S7 Table — (DOCX) [file pone.0228492.s009.docx]

S7 Table. Least square means (± upper and lower limits) of haptoglobin (mg/mL) concentrations of conditioned black Angus and black Simmental calves transported for 12 or 36 h and rested for 0, 4, 8 or 12 h^1^

|  | Treatments^2^ | | | | | | | |  |  |  |
| --- | --- | --- | --- | --- | --- | --- | --- | --- | --- | --- | --- |
| *Item* | 12-R0 | 12-R4 | 12-R8 | 12-R12 | 36-R0 | 36-R4 | 36-R8 | 36-R12 | Minimum | Maximum | *P* -value |
| LO1 | 0.1 | 0.1 | 0.1 | 0.1 | 0.1 | 0.1 | 0.2 | 0.1 | 0.25 | 0.01 |  |
| UN1 | 0.1 | 0.1 | 0.1 | 0.1 | 0.2 | 0.2 | 0.2 | 0.3 | 0.27 | 0.07 |  |
| LO2 | . | 0.1 | 0.1 | 0.1 | . | 0.2 | 0.3 | 0.4 | 0.31 | 0.11 |  |
| UN2 | 0.1 | 0.1 | 0.1 | 0.1 | 0.1 | 0.2 | 0.2 | 0.3 | 0.29 | 0.07 |  |
| 7 h | 0.1 | 0.1 | 0.1 | 0.1 | 0.2 | 0.2 | 0.3 | 0.3 | 0.32 | 0.09 |  |
| 2 d | 0.1 | 0.3 | 0.2 | 0.2 | 0.3 | 0.3 | 0.4 | 0.6 | 0.47 | 0.14 |  |
| 28 d | 0.1 | 0.2 | 0.0 | 0.1 | 0.1 | 0.2 | 0.2 | 0.1 | 0.35 | -0.05 |  |

Scheffe *P*-values are presented in the table, however, superscripts correspond to Bonferroni adjusted *P*-values for comparisons of interest. ^ab^ superscripts indicate differences between the R12 group. Least square means within a row with differing superscripts differ (*P* ≤ 0.05).

^1^Values in the table represent the mean of haptoglobin concentrations for each treatment at LO1, UN1, LO2, UN2, 7 h, 2 and 28 d.

^2^ Transport: 12: 12 h of transportation and 36: 36 h of transportation. Rest stop: R0: 0 h of rest, R4: 4 h of rest, R8: 8 h of rest and R12: 12 h of rest.
