# Supplementary material for: Effect of transport and rest stop duration on the welfare of conditioned cattle transported by road
Source: PLoS One. 2020 Mar 2;15(3):e0228492. doi: 10.1371/journal.pone.0228492 (PMC7051828; doi:10.1371/journal.pone.0228492)
Supplement: S8 Table — (DOCX) [file pone.0228492.s010.docx]

S8 Table. Least square means (± upper and lower limits) of granulocyte cell count (10^3^/µl) of conditioned black Angus and black Simmental calves transported for 12 or 36 h and rested for 0, 4, 8 or 12 h^1^

|  | Treatments^2^ | | | |  |  |  |
| --- | --- | --- | --- | --- | --- | --- | --- |
| *Item* | R0 | R4 | R8 | R12 | Maximum | Minimum | *P*-value |
| LO1 | 1.2 | 1.4 | 1.1 | 1.1 | 1.97 | 0.45 | 0.42 |
| UN1 | 4.8 | 4.8 | 4.4 | 4.1 | 5.54 | 3.53 | 0.24 |
| LO2 | - | 3.6 | 2.2 | 2.8 | 3.72 | 1.98 | 0.01 |
| UN2 | 3.6 | 3.8 | 4.5 | 5.2 | 5.23 | 3.34 | 0.16 |
| 7 h | 2.7^b^ | 5.0^a^ | 5.0^ab^ | 3.7^ab^ | 5.06 | 3.15 | <0.01 |
| 2 d | 1.8 | 2.2 | 2.9 | 3.3 | 3.56 | 1.51 | 0.31 |
| 28 d | 2.1 | 2.1 | 2.1 | 2.1 | 3.03 | 1.20 | 1.00 |

Scheffe *P*-values are presented in the table, however, superscripts correspond to Bonferroni adjusted *P*-values for comparisons of interest. ^ab^Least square means within a row with differing superscripts differ (*P*  ≤ 0.05).

^1^Values in the table represent the mean granulocyte cell count for each treatment at LO1, UN1, LO2, UN2, 7 h, 2 and 28 d.

^2^ Rest stop: R0: 0 h of rest, R4: 4 h of rest, R8: 8 h of rest and R12: 12 h of rest.
